# Supplementary material for: Healthcare worker attendance during the early stages of the COVID-19 pandemic: A longitudinal analysis of fingerprint-verified data from all public-sector secondary and tertiary care facilities in Bangladesh
Source: J Glob Health. 2020 Sep 4;10(2):020509. doi: 10.7189/jogh.10.020509 (PMC7568346; doi:10.7189/jogh.10.020509)
Supplement: Online Supplementary Document [file jogh-10-020509-s001.pdf]

Appendix Figure 1: Unsmoothed (Unadjusted) Trends in Weekly Attendance Rates Among Doctors, Nurses, and Other Staff at All Public-Sector Secondary and Tertiary Care Facilities in Bangladesh, by Cadre.

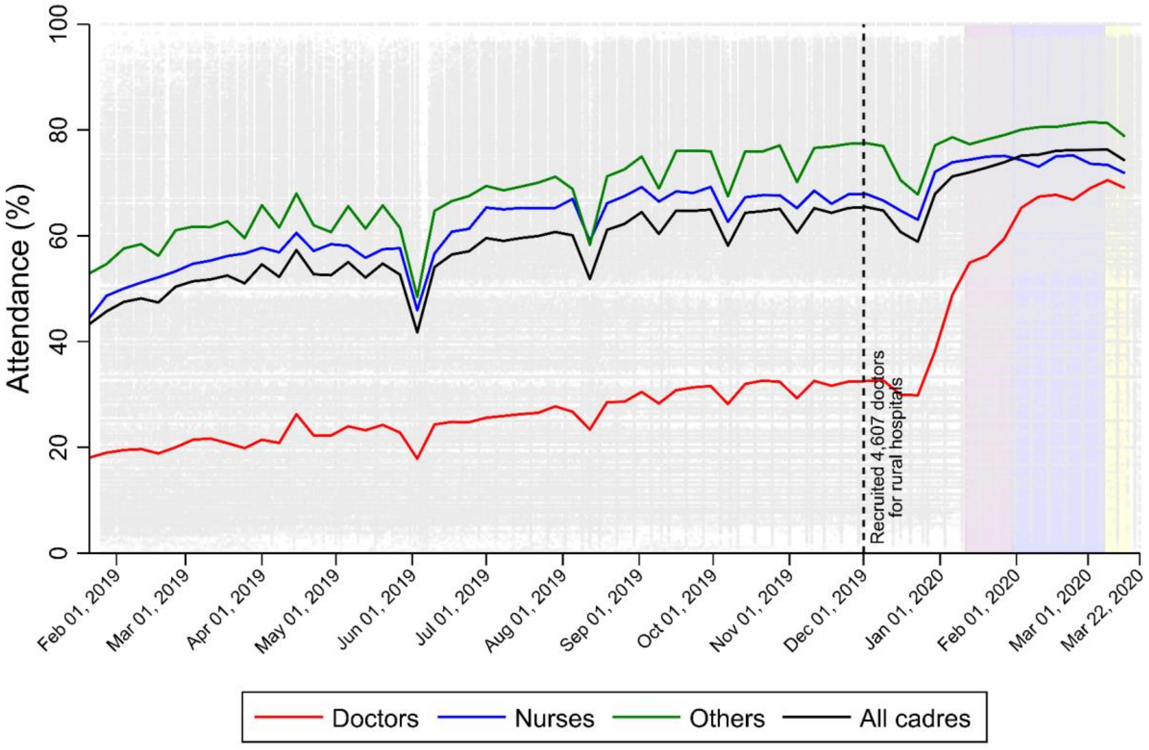

Note: 186,967 observed daily data points from 527 hospitals in 2019-2020 for doctors, nurses, and other staff are plotted in grey. Shaded areas represent the China-focused period (purple), international spread period (blue), and local spread period (yellow).
